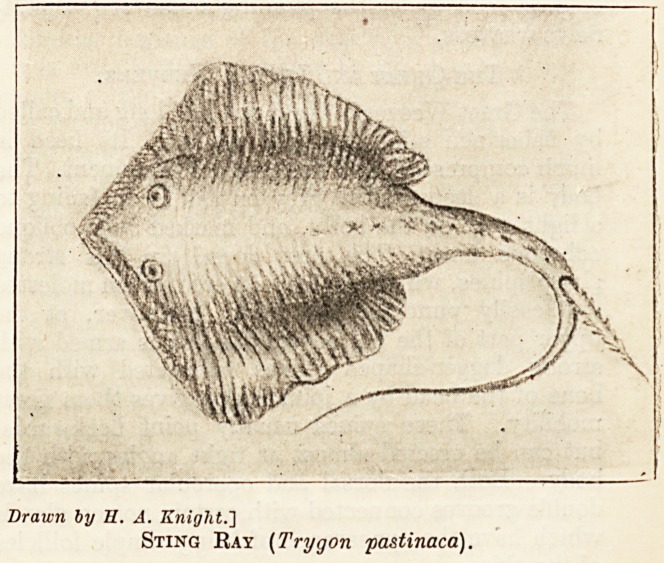# Venomous British Fishes

**Published:** 1909-02-06

**Authors:** Edward Knight


### SPECIAL ARTICLE.

### VENOMOUS BRITISH FISHES.

By EDWARD KNIGHT, L.R.C.P., L.R.C.S. Edin.

It is curious that while so much has been written in our language on snake-bites there has been comparatively little placed on record concerning the stings of fishes. Snake-bites are rare in this country, but fish-stings are very common, especially among fishermen and fishmongers.

The fishes that most often sting us are the Great and Little Weevers. In Whitechapel the former are sold as food under the punning name of "Spitalfields weavers."

The Great and Little Weevers.

The Great Weever is about one foot long and called by fishermen sting-bull and sea-cat. Its head is much compressed, and the eyes are prominent. The bod, is a deep reddish grey on the back, fading to a light grey on the belly, and marked with oblique ochreous lines. The first dorsal fin has strong sharp spines, which the fish can erect when molested and easily puncture the skin. Moreover, at the upper part of the gill covers the fish is armed with strong dagger-shaped spines connected with the bone of the head by a joint which gives them great mobility. These spines usually point backwards, but can be erected almost at right angles with the body. Both the dorsal and opercular spines have double grooves connected with certain poison glands which have the appearance of being simple follicles of the skin.

The Little Weever, sting-fish, or otter-pike is seldom more than five inches long and similarly armed. It is particularly likely to wound the bare feet of paddlers and bathers, as it often lies buried in the sand with its head and dorsal spines exposed.

A prick on the hand or foot from a weever causes much swelling and hyperæmia. The skin about the puncture may become necrosed, and bullæ form containing serum or pus. If the arm is affected the inflammation may spread to the shoulder, the swelling of the whole limb being enormous. The pain is agonising, the patient often falling into a state of collapse or becoming delirious. Usually the inflammation subsides in about three days followed by desquamation.

Experiments made with the poison of the weever on animals show that it produces gangrene of the tissues and local paralysis, and also that it has a hæmolytic action.

Drawn by H. A. Knight.]

Great Weever (*Trachinus draco.*)

Fishermen generally cut off the obnoxious prickles of weevers, and in France this precaution is rendered compulsory by law. Acting on the principle that "like cures like," they frequently apply the liver of a weever to any wound received from it.

A more rational treatment is carefully to cleanse the wound with antiseptics and extract any bit of spine left in it and then dress it with boracic lint.

Later on, if suppuration occurs, any fluctuating spot should be incised and boracic poultices applied. Applications of iodine and of turpentine are well spoken of as antidotes. Ammonia and alkalies seem unsatisfactory. To counteract the tendency to collapse, brandy or ether may be necessary, given by the mouth or injected under the skin.

### Sea-Scorpions and Father Lashers.

Two ugly little cottoid-fishes often left on our shores by the retiring tide are the Sea-Scorpion and Father Lasher. Their heads and pectoral fins are very large in proportion to their bodies.

*Drawn by H. A. Knight.*]
Gemmeous Dragonet (*Callionymus Cyra*).

The Sea-Scorpion is so called on account of the sharp spines with which its head is armed. The rays of the dorsal and pectoral fins are also distinctly sharp. This fish is seldom more than eight inches in length; its body is a rich purple brown mottled with a warm red hue.

The Father Lasher is rather longer, its colour a little lighter, and can be easily distinguished from the other species by the greater length of its spines, of which there are two on the back of the head, four on the præoperculum, three on the operculum, besides those on the snout and other parts of the body. When provoked, it puffs out its cheeks and gill-covers in a menacing manner, so as to present the sharp spines in all directions like the quills of an angry porcupine.

A fisherman dreads to be pricked by either of these two fishes. According to Dr. Bottard, a French observer, both varieties possess poison organs analogous to those of the weaver, but not so well developed.

### Dragonets and Fire Flaires.

A lovely little fish is the Gemmeous Dragonet, so called from its brilliant gem-like colours and the dragon-like aspect of its body and fins. In length it is about ten inches, and its colour golden-yellow, variegated with spots and streaks of sapphire. The first ray of the first dorsal fin is extraordinarily long; the pectorals are rounded and triangular. It is a fish not commonly seen, since it frequents deep water, and, compared with the fishes previously described, it is not markedly venomous. There are sharp spines, however, on its gill-covers, a prick from which on the hand causes acute pain, often followed by a whitlow.

In the blood of the Sharp-nosed Eel, the Conger and Muræna, there is a powerful poison, and their bites are apt to prove injurious. The Muræna has a most effective poison-injecting apparatus placed in its palate. This consists of a gland having a reservoir divided into several secondary chambers furnished with cells which secrete the poison, and three or four strong teeth; but these are not perforated by a central canal, like the teeth of some venomous snakes.

The Fire Flaire, a species of sting-ray, is sometimes caught in British waters. It bears on its tail a long bi-serrated spine which can inflict severe lacerations. The mucus secreted from the surface of the body and inoculated by the jagged spine evidently possesses poisonous properties causing much pain in the wounded part and inflammation and swelling ending not rarely in gangrene. In shape and size the fish is not unlike a skate, and its colour when cut open a bright red, hence, perhaps, its name. Sting-rays are very common in some of our Colonies. It is said to be unwise to wade out barefoot in Australian shallows as sting-rays often lie in pits in the sand. A robust man wounded by a sting-ray in Demerara died in convulsions.

Poison organs are more common in fishes than was formerly believed, and we much need more information regarding the nature of the poison they secrete, as well as stronger antidotes. It is especially during the breeding season that stinging-fish are most venomous. A fishmonger in Gravesend states that he always uses some "green mignonette" to allay the stings of fishes. This is a local name for the Emp. melilotus of the London Pharmacopœia, 1744, and appears to be nothing more than a mild emollient.

*Drawn by H. A. Knight.*]

Sting Ray (*Trygon pastinaca*).

## Figures and Tables

**Figure f1:**
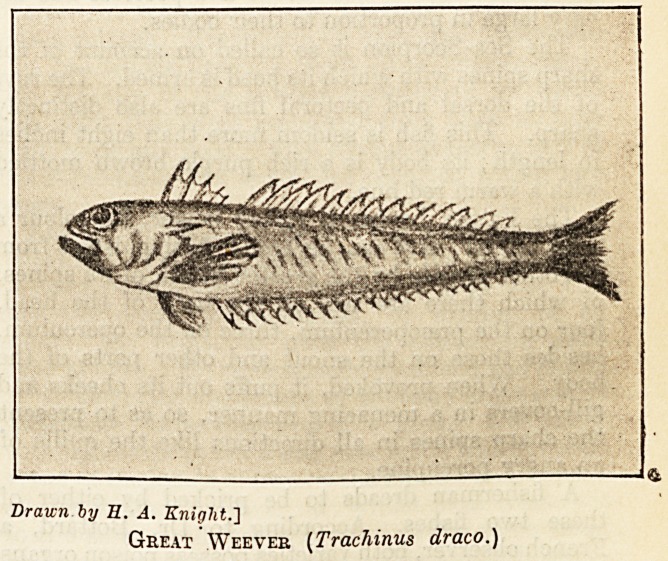


**Figure f2:**
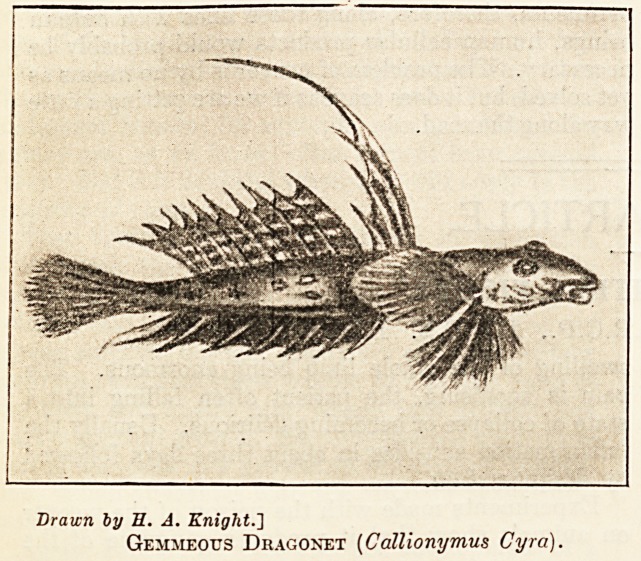


**Figure f3:**